# Heterogeneity in kinesiophobia among postoperative aortic dissection survivors: associations with exercise self-efficacy, family support, and coping styles

**DOI:** 10.3389/fpsyg.2026.1800841

**Published:** 2026-05-21

**Authors:** Xin Yang, Yuan Luo, Dan Wang

**Affiliations:** 1 Department of Cardiovascular Surgery, The First Affiliated Hospital of Chongqing Medical University, Chongqing, China; 2 Department of Cardiothoracic Surgery, Chongqing Emergency Medical Center, Chongqing, China; 3 Department of Peripheral Vessel, The First Affiliated Hospital of Xi'an Jiaotong University, Xi'an, China

**Keywords:** aortic dissection, coping style, cross-sectional study, exercise self-efficacy, family support, heterogeneity, kinesiophobia, latent profile analysis

## Abstract

**Background:**

Early postoperative kinesiophobia in aortic dissection survivors is associated with reduced exercise adherence, worsening physical and mental health, and diminished quality of life. This study aimed to identify kinesiophobia subtypes 1 month after surgery and to explore the associative pathways linking exercise self-efficacy, family support, coping styles, and kinesiophobia, thereby providing a theoretical basis for the future development of targeted interventions.

**Methods:**

This cross-sectional study enrolled 309 aortic dissection survivors (September 2024–July 2025) from three university hospitals 1 month post-surgery. Data were collected using a general information questionnaire, the Tampa Scale for Kinesiophobia Heart, the Self-Efficacy for Exercise Scale, the Family Apgar Index, and the Medical Coping Modes Questionnaire. Latent profile analysis identified kinesiophobia subtypes, followed by testing a chain mediation model integrating exercise self-efficacy, family support, coping styles, and kinesiophobia.

**Results:**

Of the 309 patients, 73.14% (*n* = 226) reported kinesiophobia. These patients were categorized into three subtypes: Subtype A (25.2%), Subtype B (17.7%), and Subtype C (57.1%). Participants with older age, Stanford Type A dissection, no spouse, lower education, and high invasive surgery correlated with higher fear. Exercise self-efficacy demonstrated a direct negative association with kinesiophobia, accounting for 38% of the total effect (−0.149, 95% CI: −0.237 to −0.081). Family support and adaptive coping style mediated this relationship by 45% (−0.178, 95% CI: −0.250 to −0.123) and 12% (−0.049, 95% CI: −0.089 to −0.023), respectively, with a significant but modest chain mediation effect of 5% (−0.019, 95% CI: −0.044 to −0.007).

**Conclusion:**

Aortic dissection survivors exhibit high kinesiophobia with marked phenotypic heterogeneity. The relationships between exercise self-efficacy, family support, coping styles, and kinesiophobia must be strictly interpreted as associative rather than causal. While these pathways underscore potential intervention targets, they remain strictly hypothesis-generating. These findings provide a roadmap for future trials to validate before clinical translation and inform risk-stratified screening by identifying high-risk phenotypes (Subtype C) and the dominant role of family support.

## 1 Introduction

Aortic dissection (AD) is a life-threatening cardiovascular emergency. According to the latest American Heart Association report, the incidence rate is 16.3 per 100,000 in males and 9.1 per 100,000 in females (Tsao et al., 2023). In China, the incidence is estimated at 5–10 per 100,000 (Zhang et al., 2025). If left untreated, AD carries a mortality risk of over 60% (Wang, 2021). Postoperative exercise rehabilitation is critical for improving quality of life (Townsend et al., 2015). Yet, AD patients frequently develop kinesiophobia—a pathological fear of movement due to excessive worry about potential injury or exacerbation of existing conditions (Gao et al., 2025). This condition contributes to muscle atrophy, reduced cardiopulmonary function (36% decline post-open repair), and increased risks of thrombosis and arteriosclerosis (Hou and Wu, 2021; Hornsby et al., 2020), significantly delaying recovery. Despite 63% of cardiologists advocating moderate aerobic exercise to mitigate aortic risks (Chaddha et al., 2015a), current rehabilitation adherence remains suboptimal, with studies highlighting reduced physical activity and compromised functional outcomes (Huang et al., 2025; Luo et al., 2022). Early psychological and behavioral intervention targeting fear-avoidance beliefs and tailored exercise protocols are urgently needed to address this critical gap.

Notably, AD exhibits distinct clinical and pathophysiological characteristics that differentiate it from other cardiovascular diseases, warranting specific investigation. Unlike chronic ischemic heart disease, AD typically presents with an abrupt, traumatic onset, precipitating acute psychological distress. The high mortality risk renders patients particularly susceptible to catastrophic cognitions regarding physical exertion. Furthermore, surgical management of AD frequently necessitates open-chest procedures, which lead to greater postoperative pain and somatic fear compared to minimally invasive interventions. These factors may interact to establish a vicious cycle: the initial trauma may undermine exercise self-efficacy, the life-threatening nature of the disease may distort perceptions of family support, and the substantial morbidity associated with open-chest surgery may exacerbate kinesiophobia. Understanding these mechanisms within the specific context of AD is therefore essential.

Exercise self-efficacy (ESE), defined as an individual’s confidence in their capability to effectively manage environmental challenges, physiological constraints, and social support systems during physical activity engagement, has emerged as a critical determinant of sustained exercise adherence (Shi, 2024). Accumulating evidence underscores its pivotal role in behavioral maintenance, with robust correlations observed between ESE levels and exercise compliance across diverse clinical populations, including stroke survivors and hypertensive patients (Shi, 2024; Zhu et al., 2022). Notably, interventional strategies targeting ESE enhancement have demonstrated reductions in exercise-related fear among peritoneal dialysis patients and rheumatoid arthritis populations (Cui et al., 2024; Sun et al., 2024). Despite these advances, the mechanism through which ESE modulates postoperative exercise anxiety in AD remains underexplored. This knowledge gap underscores the imperative for studies elucidating the roles of fear-avoidance beliefs and physiological reactivity in this vulnerable population.

Family support is a multidimensional construct. It is crucial to distinguish between perceived family support (the subjective sense of being cared for) and received family support (objective supportive behaviors provided) (Bruhn and Philips, 1984). In this study, we focus on perceived support, as it is a stronger predictor of health outcomes than the mere receipt of assistance (Dunkel-Schetter and Bennett, 1990). This perceived support significantly influences patients’ exercise adherence through emotional reinforcement and practical assistance (Feng et al., 2022). Qualitative data indicate postoperative AD patients report heightened exercise-related fear, citing unmet emotional/spiritual needs from both relatives and healthcare providers as a barrier to recovery (Breel et al., 2023). However, the specific mechanisms by which family support alleviates postoperative fear-avoidance behaviors in AD populations remain insufficiently understood.

Limited research explores how coping styles influence kinesiophobia. Psychologically, coping strategies mediate stress responses and health outcomes, with adaptive coping enhancing health behavior persistence. However, patients with cardiovascular disease often react to adverse events by avoiding exercise (Cui and Lei, 2019). This negative psychological state as a risk factor for controlling behavior severely limits patients’ participation in exercise rehabilitation. Though proactive responses to heart failure correlate with better rehabilitation adherence (Chaves and Park, 2016), the mechanism behind this association remains poorly understood in postoperative AD patients.

We propose that these factors work in a sequential manner. According to Bandura’s Social Cognitive Theory (Bandura, 1986), patients with high ESE are more likely to seek out and use external resources, including family support. The Health Action Process Approach (HAPA) model (Schwarzer, 2016) suggests that perceived family support can act as a volitional mediator, helping patients shift from fear-based avoidance to more adaptive coping strategies (Lazarus and Folkman, 1984). Based on this integrated view, we hypothesize a chain: higher ESE leads to stronger perceived family support, which promotes adaptive coping, and in turn reduces kinesiophobia.

Building upon this integrated sequential framework, this study moves beyond traditional pairwise analyses to address critical gaps in the literature. Accordingly, this study had four aims: (1) to determine the prevalence and severity of early postoperative kinesiophobia among survivors of AD; (2) to identify distinct subgroups with this condition; (3) to identify high-risk populations for early postoperative kinesiophobia; and (4) to explore the potential pathways underlying the association between ESE and kinesiophobia, particularly by examining the sequential mediation model (SEM) of “ESE → family support → adaptive coping → kinesiophobia”. These findings aim to provide novel insights for optimizing exercise rehabilitation strategies and improving patients’ quality of life.

## 2 Materials and methods

### 2.1 Design and ethics

This multicenter cross-sectional, quantitative survey strictly followed the Declaration of Helsinki and was approved by the Ethics Committee of The First Affiliated Hospital of Chongqing Medical University dated on September 23, 2024 (Approval number: ZZ2024-202-01). Written informed consent was obtained from all participants.

### 2.2 Participants

The 2024 European Society of Cardiology Guidelines for Peripheral Arterial and Aortic Diseases recommend a 1-month postoperative follow-up for AD patients (Mazzolai et al., 2024) to facilitate early clinical evaluation and timely intervention. This study enrolled 309 AD patients undergoing 1-month postoperative follow-up from September 2024 to July 2025 across three tertiary hospitals in Chongqing and Xi’an, China, using a convenience sampling method. It is important to acknowledge that this sampling strategy inherently favors patients with higher health literacy and better access to specialized care. Consequently, the sample may lack socioeconomic and geographic heterogeneity, potentially excluding rural populations or those with limited healthcare access. Inclusion criteria: Age ≥18 years; Confirmed AD diagnosis via computed tomography angiography or magnetic resonance imaging, adhering to the Stanford classification system (Professional Committee of Great Vessel Surgery Cardiovascular Surgery Branch of Chinese Medical Doctor Association, 2017); discharged smoothly without postoperative complications (e.g., organ ischemia, or surgical site infection); in stable condition, with clear consciousness, and capable of participating in exercise rehabilitation treatment; confirmed by informed consent. Exclusion criteria: Pre-existing psychiatric disorders, severe cognitive impairment, or sensory deficits (hearing/speech); Uncontrolled cardiovascular comorbidities (e.g., unstable angina, decompensated heart failure, or recent revascularization for coronary artery disease); Systemic diseases affecting mobility (e.g., severe arthritis, neuromuscular disorders) or contraindications to exercise testing.

Guided by conventional SEM guidelines recommending an observer-to-latent variable ratio of 5:1 to 20:1 (Bentler and Chou, 1987), we projected a required sample size of 195 to 260 based on a conservative 15:1 to 20:1 ratio across our 13 observed variables. Accounting for an anticipated 10% attrition rate, our recruitment target was set at 215 to 286 subjects. Ultimately, 309 participants were recruited, yielding a final analytical sample of 226 for the SEM—well within the threshold for robust statistical power.

Crucially, given that this study sought to elucidate the psychological mechanisms underlying kinesiophobia, our primary SEM analysis was restricted to patients screening positive for clinically significant fear. This focused approach was necessitated by the risk of floor effects; including asymptomatic patients would have severely restricted the variance of key psychological constructs (e.g., ESE and adaptive coping), potentially masking the very mediational pathways under investigation. Thus, while the sample size guarantees robust power, our findings are intrinsically bounded to this high-risk subgroup and should not be generalized to the broader AD population without such symptoms.

### 2.3 Investigation tools

#### 2.3.1 General information questionnaire

Through extensively reviewing literature and consulting professional clinical care experts, investigators designed a questionnaire that consists of both general sociodemographic information (e.g., gender, age, level of education, marital status, work status, monthly household income per capita, medical payment methods, tobacco history, and alcohol consumption history), as well as disease-related data (such as body mass index, disease classification, surgical methods, surgical history, and types of chronic disease combinations).

#### 2.3.2 Tampa Scale for Kinesiophobia heart (TSK-SV-heart)

It was originally adapted in Sweden by Bäck et al. (2013) from the Tampa Scale of Kinesiophobia for use with patients suffering from chronic pain. The present research adopted the Chinese scale version, which was translated (Lei et al., 2019), to measure the severity of patients’ fear of exercise-related injury or re-injury (i.e., kinesiophobia). It contains four domains—perceived danger, exercise fear, exercise avoidance, and functional impairment—assessed with 17 items in total. Items were rated on a 4-point Likert scale ranging from 1 (strongly disagree) to 4 (strongly agree), with items 4, 8, 12, and 16 reverse-scored. Total scores range from 17 to 68, with higher scores indicating greater kinesiophobia; a score of >37 suggests the presence of kinesiophobia. Cronbach’s *α* in this study was 0.890.

#### 2.3.3 Self-efficacy for exercise scale (SEE)

ESE was measured using the scale developed by Linton and Shaw (2011), and its Chinese version was translated and validated for reliability and validity by Zhang et al. (2016), to assess the degree of patient’s confidence in engaging in exercise under various circumstances. The scale consists of nine items rated on an 11-point scale from 0 (no confidence) to 10 (full confidence). The total ESE score is calculated as the mean of all item scores, with higher values reflecting greater self-efficacy. Cronbach’s *α* for this scale was 0.945.

#### 2.3.4 Family APGAR index (APGAR)

Family support was assessed using the Family Apgar Index scale developed by Smilkstein (1978), which captures perceived functional family dynamics rather than merely cataloging objective supportive behaviors. It comprises five dimensions—adaptability, partnership, growth, affection, and resolve—assessed with a single item each. Items were rated on a 3-point Likert scale ranging from 0 (hardly ever) to 2 (almost always). The total score is the sum of the scores for the five dimensions, with a maximum of 10. Higher scores indicate greater perceived family support. Based on the total score, family functioning was categorized as good (7–10), moderately impaired (4–6), or severely impaired (0–3). In the current study, Cronbach’s *α* was 0.895.

#### 2.3.5 Medical coping modes questionnaire (MCMQ)

This questionnaire, designed by Feifel et al. (1987), was translated into Chinese and adapted by Shen and Jiang (2000) to evaluate patients’ coping styles under several situations. The scale comprises three dimensions—confrontation, avoidance, and resignation—assessed with 20 items in total. Items were rated on a 4-point Likert scale, with items 1, 4, 9, 10, 12, 13, 18, and 19 reverse-scored. Total scores for each dimension were calculated independently, with higher scores indicating a stronger inclination toward that specific coping mode. In this study, Cronbach’s α coefficients for the three dimensions were 0.961, 0.958, and 0.946, respectively. Importantly, the ‘Confrontation’ subscale was defined not as non-compliance with medical advice, but as a disposition toward active problem-solving and seeking information, consistent with the construct of ‘adaptive coping’ in the context of chronic illness management (Feifel et al., 1987).

### 2.4 Data collection and quality control

Three researchers involved in this study received unified methodological training in bias mitigation strategies. The entire research process was conducted under the guidance of a senior medical graduate supervisor, and a pilot survey was carried out in advance at three university-affiliated hospitals to minimize bias in data collection to the greatest extent possible. Paper-based questionnaires were administered on-site and collected during the 1-month postoperative outpatient follow-up. The first patient’s data was collected on September 24, 2024. Researchers provided standardized verbal and written instructions to participants, explicitly explaining the study’s objectives, significance, and precautions while avoiding leading questions or suggestive language. Informed consent was obtained from all participants before they completed the questionnaires anonymously. For those unable to complete them independently due to postoperative limited upper limb mobility or low educational level, the investigators offered assistance in filling them out. After completion, researchers conducted on-site inspections of the questionnaires, promptly verifying and resolving any questionable responses. Maintaining blinded data entry to prevent observer bias. A total of 323 questionnaires were distributed, with 309 valid responses returned (95.67% response rate).

### 2.5 Statistical analysis

SPSS 25.0 software was used for descriptive statistics, chi-square tests, and Pearson correlation analysis of the data. Measurement data that met the criteria for a normal distribution were expressed as mean ± standard deviation (x ± s). Data with an absolute skewness value < 3 and an absolute kurtosis value < 10 are considered to be an approximate normal distribution. Enumeration data were expressed as frequency and percentage. The analyses of latent categories, SEM, and mediating effects were conducted using Mplus 8.3 software. Latent profile analysis (LPA) identified kinesiophobia categories, with model fit goodness assessed using Akaike Information Criterion (AIC), Bayesian Information Criterion (BIC), Sample-Size-Adjusted Bayesian Information Criterion (aBIC), and Entropy. The optimal model was selected based on Lo–Mendell–Rubin (LMR) and Bootstrap likelihood ratio test (BLRT) significance. SEM was used to estimate the pathway relationships between exercise self-efficacy, family support, medical coping styles, and kinesiophobia. The mediating effect was tested using Bootstrap with repeated sampling 5,000 times. The effect value with 95% confidence intervals (Bootstrapped Lower Level Confidence Interval, BootLLCI; Bootstrapped Upper Level Confidence Interval, BootULCI) was calculated. If the interval excludes 0, the mediating effect is statistically significant. All statistical data were examined using two-tailed tests, with the value of *p* < 0.05 being statistically significant.

It is important to note that all variables were assessed 1 month post-surgery, and the relationships among them were subsequently modeled using SEM. Consequently, the proposed ‘sequential’ mediation model represents a theoretically hypothesized chain based on the Social Cognitive Theory, rather than an empirically verified temporal sequence.

## 3 Results

### 3.1 Incidence and latent class profiles of kinesiophobia

**Figure 1:**
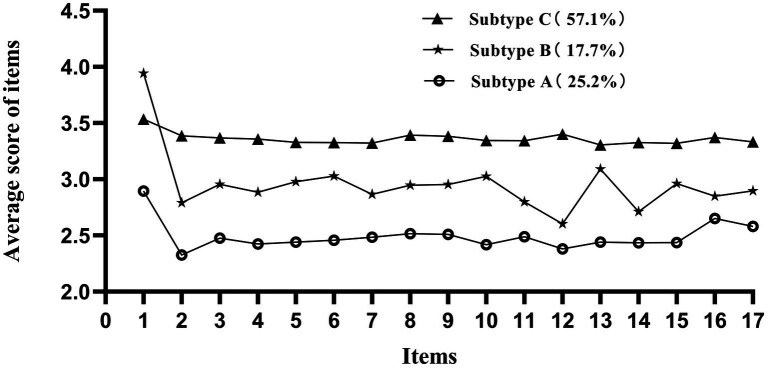
Latent profiles of kinesiophobia.

Among 309 participants with AD assessed at 1-month post-surgery, 226 (73.14%) screened positive for kinesiophobia. LPA was subsequently performed on this cohort of 226 patients, identifying five candidate models. We selected Model 3, which demonstrated superior fit indices (AIC, BIC, aBIC) and significant LMR and BLRT values. While Model 4 also yielded a significant BLRT, its non-significant LMR (*p* = 0.303) suggested no substantial improvement over Model 3. Model 3 balanced parsimony with high classification accuracy (entropy = 0.931) and clear clinical relevance. Robust subgroup separation was confirmed by high average latent class probabilities (98.5, 95.2, and 97.3%). This analysis delineated three distinct profiles: (1) Subtype A (25.2%, TSK-SV-Heart Score 38–46), characterized by minimal fear interference; (2) Subtype B (17.7%, TSK-SV-Heart Score 47–52), showing partial activity avoidance; and (3) Subtype C (57.1%, TSK-SV-Heart Score 53–65), exhibiting severe functional impairment, as shown in Table 1 and Figure 1.

**Table 1:** LPA models of kinesiophobia after AD surgery (*n* = 226).

Model	AIC	BIC	aBIC	Entropy	P(LMR)	P(BLRT)	Category probability (%)
1	7768.319	7884.618	7776.863	-	-	-	-
2	6844.208	7022.076	6857.276	0.952	0.0000	0.0000	31.9/68.1
3	6722.735	6962.173	6740.326	0.931	0.0310	0.0000	25.2/17.7/57.1
4	6471.512	6772.519	6493.626	0.976	0.3030	0.0000	25.2/26.5/22.6/25.7
5	6448.019	6810.596	6474.656	0.977	0.7686	0.0000	25.2/19.5/3.1/26.5/25.7

AIC, Akaike Information Criterion; BIC, Bayesian Information Criterion; aBIC, Sample-Size-Adjusted Bayesian Information Criterion; LMR, Lo–Mendell–Rubin Likelihood Ratio Test; BLRT, Bootstrap Likelihood Ratio Test.

### 3.2 Demographic differences in different profiles of kinesiophobia

Chi-square tests showed that patients who were older, without a spouse, less educated, diagnosed with Stanford type A dissection, and underwent more invasive surgical procedures were more likely to be classified into the high-fear profile, as shown in Table 2.

**Table 2:** Comparison of demographic differences between different profiles of kinesiophobia *n* (%).

Item		Subtype A (25.2%)	Subtype B (17.7%)	Subtype C (57.1%)	Total	χ^2^	*P*
Gender	Male	43 (75.44)	32 (80.00)	96 (74.42)	171 (75.66)	0.519	0.772
	Female	14 (24.56)	8 (20.00)	33 (25.58)	55 (24.34)		
Age	< 25 years old	1 (1.75)	0 (0.00)	0 (0.00)	1 (0.44)	99.973	0.000**
	25–35 years old	23 (40.35)	1 (2.50)	0 (0.00)	24 (10.62)		
	36–45 years old	19 (33.33)	4 (10.00)	21 (16.28)	44 (19.47)		
	46–55 years old	6 (10.53)	16 (40.00)	34 (26.36)	56 (24.78)		
	> 55 years old	8 (14.04)	19 (47.50)	74 (57.36)	101 (44.69)		
BMI	< 18.5	5 (8.77)	0 (0.00)	6 (4.65)	11 (4.87)	4.228	0.376
	18.5 ~ 23.9	26 (45.61)	19 (47.50)	56 (43.41)	101 (44.69)		
	≥ 24	26 (45.61)	21 (52.50)	67 (51.94)	114 (50.44)		
Marital status	With a spouse	55 (96.49)	38 (95.00)	81 (62.79)	174 (76.99)	34.243	0.000**
	Without a spouse	2 (3.51)	2 (5.00)	48 (37.21)	52 (23.01)		
Work status	Retired	11 (19.30)	5 (12.50)	33 (25.58)	49 (21.68)	5.453	0.708
	Unit employed	17 (29.82)	13 (32.50)	29 (22.48)	59 (26.11)		
	Self-employed	7 (12.28)	5 (12.50)	12 (9.30)	24 (10.62)		
	Peasant	15 (26.32)	12 (30.00)	42 (32.56)	69 (30.53)		
	Unemployed	7 (12.28)	5 (12.50)	13 (10.08)	25 (11.06)		
Education	Junior high school or below	2 (3.51)	22 (55.00)	76 (58.91)	100 (44.25)	118.131	0.000**
	Senior high/polytechnic school	7 (12.28)	10 (25.00)	39 (30.23)	56 (24.78)		
	Junior college	14 (24.56)	4 (10.00)	11 (8.53)	29 (12.83)		
	Undergraduate	23 (40.35)	4 (10.00)	3 (2.33)	30 (13.27)		
	Postgraduate and above	11 (19.30)	0 (0.00)	0 (0.00)	11 (4.87)		
Monthly household income per capita	< 3,000 yuan	20 (35.09)	12 (30.00)	38 (29.46)	70 (30.97)	3.297	0.509
	3,000–5,000 yuan	27 (47.37)	17 (42.50)	52 (40.31)	96 (42.48)		
	> 5,000 yuan	10 (17.54)	11 (27.50)	39 (30.23)	60 (26.55)		
Medical payment type	Commercial insurance	0 (0.00)	0 (0.00)	3 (2.33)	3 (1.33)	3.117	0.538
	Medical insurance	54 (94.74)	37 (92.50)	121 (93.80)	212 (93.81)		
	Personal payment	3 (5.26)	3 (7.50)	5 (3.88)	11 (4.87)		
Disease type	Stanford A	8 (14.04)	13 (32.50)	72 (55.81)	93 (41.15)	29.995	0.000**
	Stanford B	49 (85.96)	27 (67.50)	57 (44.19)	133 (58.85)		
Surgical approach	Prosthetic vascular replacement	15 (26.32)	3 (7.50)	3 (2.33)	21 (9.29)	34.638	0.000**
	Stent intervention	38 (66.67)	27 (67.50)	85 (65.89)	150 (66.37)		
	Prosthetic vascular replacement + stent placement	4 (7.02)	10 (25.00)	41 (31.78)	55 (24.34)		
History of smoking	No	21 (36.84)	17 (42.50)	64 (49.61)	102 (45.13)	2.739	0.254
	Yes	36 (63.16)	23 (57.50)	65 (50.39)	124 (54.87)		
History of liquor	No	26 (45.61)	20 (50.00)	71 (55.04)	117 (51.77)	1.467	0.480
	Yes	31 (54.39)	20 (50.00)	58 (44.96)	109 (48.23)		
History of surgery	No	41 (71.93)	23 (57.50)	74 (57.36)	138 (61.06)	3.787	0.151
	Yes	16 (28.07)	17 (42.50)	55 (42.64)	88 (38.94)		
Types of chronic disease	0	16 (28.07)	9 (22.50)	31 (24.03)	56 (24.78)	8.018	0.237
	1	27 (47.37)	22 (55.00)	72 (55.81)	121 (53.54)		
	2	7 (12.28)	9 (22.50)	17 (13.18)	33 (14.60)		
	3 or more	7 (12.28)	0 (0.00)	9 (6.98)	16 (7.08)		

*p < 0.05 **p < 0.01. BMI, weight (kg) / height (m)^2^, Body Mass Index.

### 3.3 Current status and correlation analyses of kinesiophobia, ESE, family support, and coping styles

The kinesiophobia score for the 226 participants was 52.15 ± 6.78. The scores for the three categories of Subtype A, Subtype B, and Subtype C were 42.37 ± 2.36, 50.08 ± 1.70, and 57.12 ± 2.95, respectively. The score of SEE was 4.53 ± 2.08; the score of APGAR was 4.38 ± 3.11; the confrontation dimension of MCMQ was 11.36 ± 6.08, avoidance dimension was 11.32 ± 5.71, and resignation dimension was 8.08 ± 4.08. Pearson correlation analysis revealed that early postoperative kinesiophobia in AD patients was significantly negatively correlated with ESE (r = −0.696), family support (r = −0.733), and the positive coping style (r = −0.618) (Table 3).

**Table 3:** Pearson correlation analyzes of kinesiophobia, ESE, family support, and coping styles.

Variables		SEE	APGAR	Confrontation	Avoidance	Resignation	TSK-SV-Heart
SEE		1					
APGAR		0.606**	1				
MCMQ	Confrontation	0.569**	0.496**	1			
	Avoidance	−0.522**	−0.593**	−0.220**	1		
	Resignation	−0.485**	−0.530**	−0.202**	0.780**	1	
TSK-SV-Heart		−0.696**	−0.733**	−0.618**	0.653**	0.595**	1

*p < 0.05, **p < 0.01. SEE, Self-Efficacy for Exercise Scale, to assess ESE; APGAR, Family Apgar Index, to assess family support; MCMQ, Medical Coping Modes Questionnaire, to assess medical coping styles; TSK-SV-Heart, Tampa Scale for Kinesiophobia Heart, to assess kinesiophobia.

### 3.4 Independent and serial mediating roles of family support and coping styles in ESE and kinesiophobia

**Figure 2:**
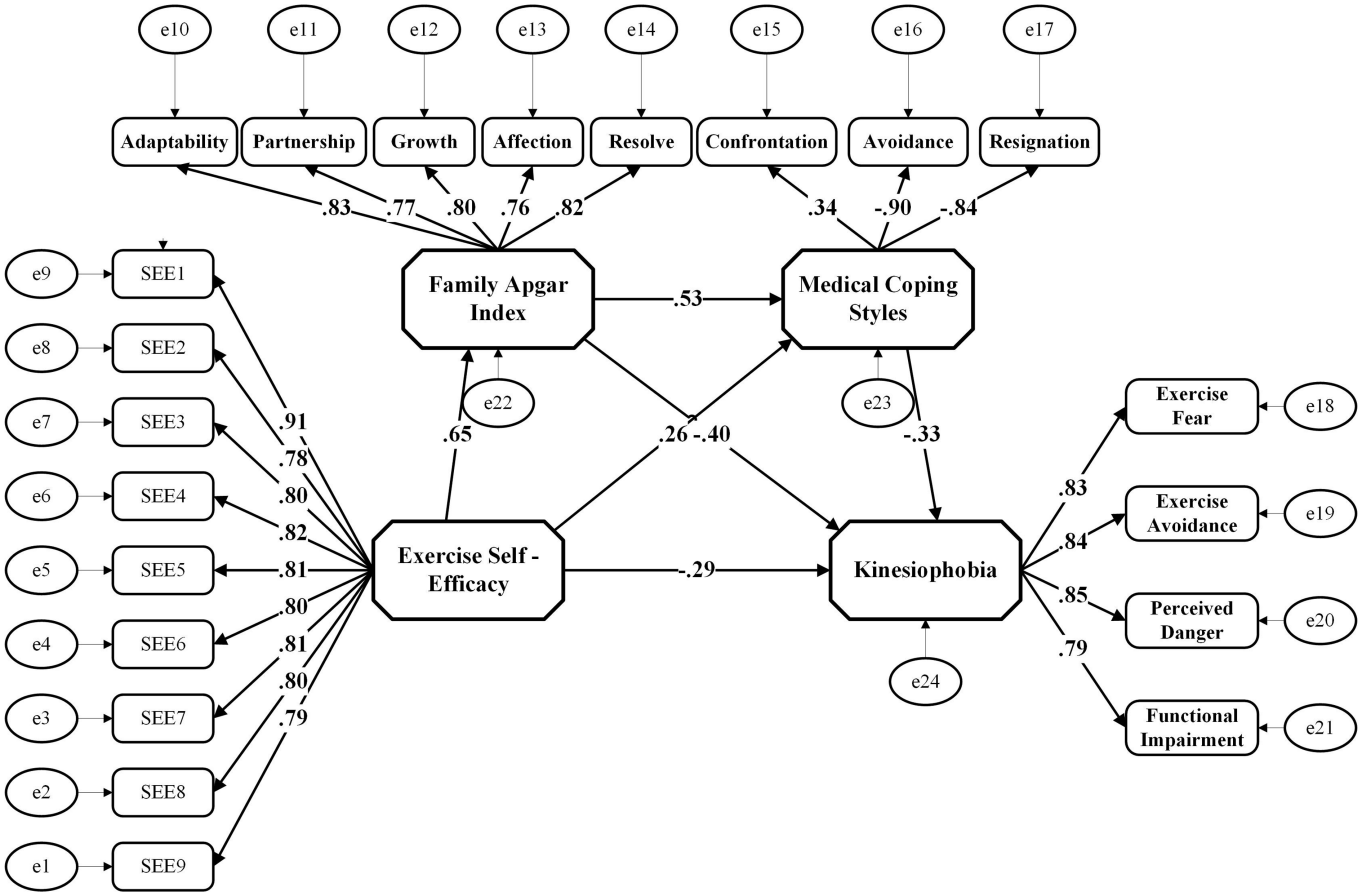
Chain mediating model of ESE and kinesiophobia.

We tested the hypothesized chain mediation using SEM in 226 patients screening positive for kinesiophobia. The model specified kinesiophobia as the outcome, ESE as the predictor, and family support and coping styles as mediators. All model fit indicators met the statistical criteria (Comparative Fit Index = 0.949 > 0.9, Tucker-Lewis Index = 0.942 > 0.9, Root Mean Square Error of Approximation = 0.067 < 0.08). The model demonstrated that ESE significantly predicted postoperative kinesiophobia through dual-mediating pathways involving family support and medical coping styles. ESE had a significant direct negative effect on kinesiophobia (*β* = −0.149, 95% CI: −0.237 to −0.081), representing 38% of the total effect. Mediation analysis revealed that ESE enhanced family support (*β* = 0.649, *p* < 0.001), which in turn fostered adaptive medical coping style (*β* = 0.250, *p* < 0.005) and reduced kinesiophobia (*β* = −0.514, *p* < 0.001). Family support and adaptive coping served as significant independent mediators, accounting for 45% (*β* = −0.178, 95% CI: −0.250 to −0.123) and 12% (*β* = −0.049, 95% CI: −0.089 to −0.023) of the total effect, respectively. The chain-mediated pathway (ESE → family support → adaptive coping → kinesiophobia) yielded a significant but modest standardized effect (*β* = −0.019, 95% CI: −0.044 to −0.007), explaining an additional 5%. The total indirect effect (−0.246, 62% of overall effect) underscored the central role of psychosocial factors in mitigating exercise avoidance (Figure 2; Tables 4, 5).

**Table 4:** Pairwise relationships among ESE, family support, coping style, and kinesiophobia.

Pathway	B	β	SE	CR	*P*	Lower	Upper
ESE → family support	0.154	0.649	0.017	9.005	***	0.123	0.189
ESE → adaptive coping	0.871	0.419	0.196	4.453	***	0.468	1.242
family support → adaptive coping	2.182	0.250	0.767	2.846	0.004	0.783	3.813
ESE → kinesiophobia	−0.149	−0.280	0.039	−3.846	***	−0.237	−0.081
adaptive coping → kinesiophobia	−0.057	−0.221	0.015	−3.695	***	−0.087	−0.026
family support → kinesiophobia	−1.152	−0.514	0.166	−6.946	***	−1.506	−0.851

***p < 0.001. B, Beta Coefficient; β, regression coefficients; SE, Standard Error; CR, Conditional Residual.

**Table 5:** Chain-mediating testing of family support and adaptive coping style on ESE and kinesiophobia.

Pathway	Effect value (95% CI)	*P*	Effect ratio (%)
ESE → kinesiophobia	−0.149 (−0.237~−0.081)	0.000	38
ESE → family support → kinesiophobia	−0.178 (−0.250~−0.123)	0.000	45
ESE → adaptive coping → kinesiophobia	−0.049 (−0.089~−0.023)	0.002	12
ESE → family support → adaptive coping → kinesiophobia	−0.019 (−0.044~−0.007)	0.027	5
Total effect	−0.395 (−0.487~−0.309)	0.000	100

CI, confidence interval.

## 4 Discussion

### 4.1 Postoperative AD patients have high levels of early kinesiophobia with significant heterogeneity

This study revealed that 1 month after AD, the incidence of kinesiophobia was 73.14%, with a score of 52.15 ± 6.78, indicating a high overall level. Patients who have experienced acute cardiovascular events, especially those with AD, have a tendency to avoid exercise (Wang, 2022). This may be attributed to the intense tearing pain and the overwhelming sense of imminent death they have experienced. As a result, these patients fear that physical activity might trigger another episode or cause further injury. This avoidance behavior serves as a self-protection mechanism they believe. A survey conducted by Chaddha et al. (2015b) involving 197 AD patients has revealed that 76% of those who underwent surgery believed that they could no longer engage in physical exercise as they did before. Sharma et al. (2022) have also confirmed that AD patients do not have high compliance with exercise rehabilitation after surgery. They may not fully recognize the potential serious consequences of avoiding exercise, such as disability or dysfunction of body organs, which could result from a lack of physical activity. A potential vicious loop may form between kinesiophobia and negative health conditions, severely reducing their quality of life post-surgery.

Additionally, LPA revealed significant heterogeneity in postoperative kinesiophobia among 226 AD patients at 1-month follow-up, classifying them into three phenotypes: Subtype A, Subtype B, and Subtype C. The Subtype C group exhibited distinct clinical characteristics: older age, being unmarried or widowed, lower education, Stanford A dissection, and invasive surgical procedures. Advanced age independently predicted elevated kinesiophobia (Gao, 2023). Older patients may experience higher levels of kinesiophobia due to several factors. They often have more underlying diseases and complications, which may weaken their physical functions and overall health status. Meanwhile, older individuals tend to have a reduced ability to acquire and process new information and new things, and their mental state may be less stable, potentially leading to increased resistance to exercise and a heightened fear of exercise. Unmarried/widowed status was associated with heightened fear. Cui and Lei (2019) found that married patients with coronary heart disease had lower kinesiophobia levels than unmarried or widowed individuals, which may be because spouses provide positive support and assistance during the post-traumatic recovery and emotional growth of patients. Family support and attitudes affect patients’ motivation and compliance with physical activity (Feng et al., 2022). Patients without a spouse may be more sensitive to the potential risks of exercise and lacking the encouragement, emotional support, and family care from a partner may be more likely to avoid physical activity. Bauer’s theoretical model of risk–benefit trade-off demonstrates the coexistence of positive and negative factors in decision-making (Zhang, 2023). In this study, patients with higher levels of education had lower levels of kinesiophobia. This may probably be because they had a stronger perception of the risk–benefit of exercise and a clearer understanding that lack of physical activity leads to an increased likelihood of secondary injury. Besides, engaging in exercise can help mitigate risk factors and improve quality of life. Education is an important predictor of kinesiophobia and physical activity levels (Zhu et al., 2024). Patients with higher levels of literacy have a better understanding and acceptance of their conditions, enabling them to access health resources more easily, thereby more likely to engage in exercise. Stanford A AD is more acute than Stanford B, with a higher mortality rate, greater involvement of multiple organs, and more severe complications. Surgery for Stanford A also involves greater trauma than Stanford B, and requires thoracotomy for prosthetic vascular replacement, while Stanford B patients need only intervention with smaller trauma. Bad experiences and treatment place a heavier psychological burden on patients, leading to greater rehabilitation challenges. They may directly associate exercise with the risk of “rupture again,” forming a false belief that “physical activity = danger”. Stanford A patients may develop a heightened fear of “exercise-induced sudden death”, making them more cautious and resistant to accepting exercise rehabilitation. However, Stanford B patients typically experience a milder condition and are treated with less invasive approaches, so they are more open to the recommendation of appropriate exercise. Patients in the Subtype B scored higher on Item 13, “When should I stop physical activity/exercise so that I don’t harm myself” compared to other items, close to the level of Subtype C. This indicated that these patients might overgeneralize danger signals, failing to recognize the positive effects of moderate exercise on cardiac rehabilitation. Their serious cognitive biases contributed to higher levels of kinesiophobia.

Reflecting the distinct phenotypic profiles outlined above, the identification of high-risk phenotypes (particularly Subtype C) supports the need for differentiated interventions. Building on the work of Bäck et al. (2016), early exercise-based cardiac rehabilitation is recommended; however, modalities must be tailored to individual patient profiles. Specifically, medical professionals could develop stratified management plans based on the LPA characteristics identified herein. To address the specific needs of these subgroups, targeted educational initiatives (such as specialized lectures and online platforms) are warranted to provide accurate rehabilitation knowledge. Moreover, for patients exhibiting high cognitive avoidance, post-traumatic cognition might be reshaped using virtual reality technology (Wang et al., 2023) or other immersive programs that simulate sports scenarios. These efforts should be complemented by robust family support and the implementation of structured, progressive exercise plans. Ultimately, future research should establish continuous monitoring and digital follow-up systems to bolster adherence and reduce kinesiophobia.

### 4.2 Current status of ESE, family support, coping styles in kinesiophobia patients 1 month after AD surgery

Postoperative AD patients exhibited low ESE (4.53 ± 2.08), similar to findings by Wen et al. (2024). The underlying reasons might relate to the involvement of AD to organs and viscera in postoperative physiological limitations such as insufficient organ function, fatigue, loss of appetite, and slow recovery of physical strength; patients may also experience a cognitive bias, excessively concerned about complications due to the belief that “exercise is dangerous”; the uncertainty of the rehabilitation process leads to confusion about appropriate exercise intensity and safety; post-traumatic anxiety caused by chest pain or a sense of near-death experience during an acute episode, can contribute to developing an avoidance mentality to exercise scenarios, and it is worsened due to the absence of personalized guidance and supervision during exercise recovery. All of these lead to patients’ lack of confidence in exercise recovery.

The Family APGAR index score was 4.38 ± 3.11, with mean scores for the five dimensions ranging from 0.83 to 0.92 (each dimension had a maximum score of 2). These results indicate moderate dysfunction and significant deficits in the patient’s family regarding crisis coping, responsibility sharing, and emotional support. The family failed to fully leverage its supportive role in improving the patient’s psychological deficits and facilitating rehabilitation.

The MCMQ scores were 11.36 ± 6.08 for confrontation, 11.32 ± 5.71 for avoidance, and 8.08 ± 4.08 for resignation, suggesting that AD patients mainly adopted confrontation or avoidance strategies. This finding is consistent with the research by Liu J. et al. (2025) on coping styles of patients with unstable angina pectoris after percutaneous coronary interventions. However, when compared with the scores for confrontation (19.48 ± 3.81), avoidance (14.44 ± 2.97), and resignation (8.81 ± 3.17) in the Chinese normal model (He et al., 2018), AD patients scored significantly lower in confrontation, indicating weaker active coping capacity and reliance on passive acceptance or avoidant strategies. This reflects weak psychological resilience, which may increase risks of poor treatment adherence and delayed recovery.

Therefore, healthcare providers should effectively assume the roles of “guides,” “supporters,” and “coordinators,” and engage in multidisciplinary team collaboration to develop and implement psychological and behavioral intervention plans for patients and their families from the perspective of enhancing cognition, behavior, and support. Outcomes should be consolidated through measures like follow-up to assist patients in overcoming kinesiophobia and achieving rehabilitation goals.

### 4.3 Relationship between ESE, family support, coping styles, and kinesiophobia in patients 1 month after AD surgery

#### 4.3.1 Association between ESE and kinesiophobia

This study explored the potential associative pathways underlying the interrelationships among ESE, family support, coping styles, and kinesiophobia in AD patients 1 month after surgery using an Mplus chain-mediating model. ESE showed a strong negative correlation (r = −0.696) with kinesiophobia, with a direct effect of *β* = −0.149, accounting for 38%, which was consistent with the findings of Liu Z. J. et al. (2025) in patients with primary liver cancer. The Health Action Process Approach proposed by Schwarzer (2016) posits that behavioral intention is a key predictor of healthy behavior. Supporting this, Wang et al. (2022) demonstrated that individuals with high self-efficacy exhibit greater confidence in exercise. They tend to be more proactive in regular physical exercise, showing better adherence to rehabilitation regimens while perceiving lower risks, which results in milder symptoms of kinesiophobia. Within this theoretical framework, our findings suggest that enhancing ESE may be a potential mechanism to alleviate kinesiophobia. Future research employing longitudinal or interventional designs is needed to determine whether structured strategies (e.g., setting ladder goals and cognitive behavioral training) could translate self-efficacy into tangible behavioral changes, thereby confirming a viable clinical pathway.

#### 4.3.2 Family support and adaptive coping style independently mediate ESE and kinesiophobia

ESE mediated postoperative kinesiophobia in AD patients through dual indirect pathways: family support and adaptive coping style, with the total indirect effect accounting for 62%.

ESE showed a robust positive correlation with perceived family support, suggesting that higher ESE fosters stronger family engagement during rehabilitation. This is likely because highly self-efficacious individuals are more willing to communicate their recovery and exercise needs, thereby eliciting greater emotional support, behavioral guidance, and material assistance. Conversely, family support exhibited a significant inverse relationship with kinesiophobia, indicating that a higher level of family support could effectively alleviate patients’ anxiety and fear during physical activity by providing emotional comfort and behavioral companionship. Specifically, the indirect pathway via family support accounted for 45% of the total effect (*β* = −0.178). Beyond merely relieving uneasiness, this positive support bolsters psychological confidence in facing exercise challenges, thereby effectively reducing the fear of exercise. This finding aligns with the core principle of Social Support Theory (Chen and Lu, 2014), which states that support from important others, such as family, can cushion the negative impact of stressful events on individuals. Building on this framework, our results point to promising clinical targets for future intervention strategies. Based on these observed associations, researchers could develop dual-level approaches: (1) enhancing ESE through cognitive-behavioral training to solidify exercise confidence, and (2) implementing family-centered programs (e.g., structured health education and co-designed rehabilitation plans) to optimize external support. We recommend that future longitudinal or intervention studies test whether optimizing these factors can effectively improve adherence to postoperative exercise regimens.

ESE showed differential correlations with medical coping styles: a strong positive association with confrontation and inverse relationships with avoidance and resignation, which is in line with the study by Li et al. (2019). This pattern suggests that high ESE fosters proactive problem-solving like adhering to rehabilitation protocols, whereas low ESE is associated with maladaptive strategies like avoidance. Notably, the three coping styles investigated in this study—confrontation, avoidance, and resignation—demonstrated different effects in predicting kinesiophobia among patients after AD surgery: While confrontation coping inversely predicted kinesiophobia (stronger active problem-solving was associated with lower fear of movement), avoidance and resignation exhibited significant positive effects on kinesiophobia. Positive coping style may offer a sense of safety and scientific guidance, helping to reduce concerns and anxieties that may arise during exercise. In contrast, the coping styles of “avoidance” and “resignation” appear to amplify the emotional perception of threat from “exercise-induced injury,” exacerbate kinesiophobia, and severely hinder the rehabilitation process. The mediation analysis revealed that ESE exhibited an indirect negative association with kinesiophobia via confrontation (indirect effect = −0.049, 12% mediation). Highly self-efficacious patients tended to actively follow clinical advice, attend regular appointments, and engage in rehabilitation training. These behaviors are likely linked to enhanced psychological safety and reduced actual risks associated with exercise on a behavioral level, thereby mitigating exercise fear. This aligns with the prediction through the Health Belief Model (Ghorbani-Dehbalaei et al., 2021) that an individual’s perception of health threats and belief in the effectiveness of interventions influence their willingness to adopt healthy behaviors. Translating these findings into practice, medical professionals could apply the “health belief” theory to help patients regain confidence in exercise following AD surgery by addressing risk assessment, exercise guidance, psychological counseling, and family support. Correcting the misconception that “exercise is dangerous”—potentially involving family supervision—may systematically enhance self-efficacy. Such approaches might facilitate a shift from passive treatment to active health management, disrupting the cycle of low ESE and high fear.

#### 4.3.3 Family support and adaptive coping style as a supplementary chain-mediating pathway

While the chain-mediating pathway (ESE → family support → adaptive coping → kinesiophobia) was statistically significant, its effect size was modest, accounting for only 5% of the total effect (*β* = −0.019). This suggests that although this sequential cascade exists, it functions primarily as a supplementary mechanism rather than a primary driver of kinesiophobia.

The finding implies that ESE may foster engagement with family members, which in turn encourages adaptive coping strategies. Consistent with prior work, family support reduces psychological burden and provides informational resources (Zhou et al., 2020; Chen, 2024), while active coping is linked to reduced uncertainty and better psychological well-being (Shi et al., 2023). However, given the small absolute magnitude of this pathway, clinicians should view it as a secondary reinforcement loop rather than the main target for intervention.

In contrast, the direct effect of family support (β = −0.514) emerged as the most potent factor. Therefore, healthcare providers should prioritize strengthening family support systems as the primary intervention strategy. While fostering adaptive coping remains beneficial, the clinical utility of targeting the complex chain pathway is limited compared to directly bolstering the patient-family bond to enhance exercise confidence and mitigate kinesiophobia.

### 4.4 Limitations

Several limitations warrant consideration. Regarding design and causality, the cross-sectional nature of this study precludes any assessment of temporal precedence. Consequently, the pathways identified herein should be interpreted as associations rather than causal mechanisms, as reverse causality (e.g., high kinesiophobia eroding perceived family support) remains plausible.

Additionally, regarding measurement and confounding, two issues require nuance. Regarding coping: While we operationalized the MCMQ’s ‘Confrontation’ subscale as active coping, we acknowledge that in a post-surgical context, a degree of ‘protective caution’ is clinically prudent. Regarding confounding: Beyond basic demographics, we lacked data on objective clinical indicators—specifically post-operative pain levels, comorbidity burden, and functional status—which could independently drive both lower self-efficacy and higher kinesiophobia. Pre-existing personality traits (e.g., neuroticism) and baseline psychological distress were also unmeasured. Furthermore, reliance on self-report instruments across all constructs introduces the risk of common method bias, potentially inflating the observed associations. Additionally, while score-based subtypes aided exploratory analysis, they lack external clinical validation and may not translate to distinct phenotypes.

Finally, regarding sampling, convenience recruitment from tertiary urban hospitals limits generalizability. This approach likely selected for patients with higher health literacy, potentially underestimating the impact of financial toxicity or transportation barriers faced by rural populations. The buffering effect of family support observed here might therefore be attenuated in socioeconomically diverse settings.

Building on these limitations, future studies should move beyond cross-sectional snapshots. Prospective longitudinal designs are needed to track patients across recovery stages (e.g., 1, 3, and 6 months), clarifying how these pathways unfold over time. Multi-center collaborations ought to employ stratified sampling to capture rural and low-income populations, testing whether family support buffers fear equally across socioeconomic strata. Methodologically, integrating qualitative methods like cognitive interviewing would help distinguish ‘necessary caution’ from ‘maladaptive avoidance,’ providing the external validation our current subtypes lack.

## 5 Conclusion

One month after AD surgery, most patients exhibited high levels of kinesiophobia, with significant heterogeneity observed. Advanced age, no spouse, low educational attainment, Stanford A disease type, and undergoing more invasive surgical procedures were associated with a higher likelihood of severe kinesiophobia.

The relationship between ESE and kinesiophobia was characterized by complex associative linkages, involving both direct effects and indirect pathways through family support and coping styles. Notably, the data suggest that merely enhancing individual self-efficacy may be insufficient to fully mitigate fear; rather, the interplay between social support and behavioral strategies appears relevant.

Interpreted strictly as hypothesis-generating, these findings highlight potential targets for future investigation. However, the cross-sectional design precludes the formulation of definitive clinical recommendations. Future RCTs are warranted to validate whether interventions addressing these specific pathways can effectively reduce kinesiophobia and improve patient outcomes.

## Data Availability

The datasets presented in this study can be found in online repositories. The names of the repository/repositories and accession number(s) can be found in the article/supplementary material.
